# (Gluzinskt’s) Method for Determining the Character of a Pyloric Stenosis with Especial Reference to the Transition of the Round Ulcer into Cancer

**Published:** 1913-01

**Authors:** 


(GLUZINSKI'S) METHOD FOR DETERMINING THE CHARACTER OF A PYLORIC STENOSIS WITH ESPECIAL REFERENCE TO THE TRANSITION OF THE ROUND ULCER INTO CANCER. The method in question was first described in detail by the author in the *Gazeta Leraska* (1901 Warsaw, Russia) and in Vol. X of the *Mitteilungen Aus den Grenzgebienten der Medizin und Chirurgie.*

Its object was the early recognition of the character of a pyloric stenosis, i. e.: Whether it is caused by a round ulcer or cancer and eventually whether one is dealing with just a simple ulcer, or an ulcer becoming malignant.

Principal conclusions detailed in former works:

(1) Ninety per cent of gastric ulcers are situated in the pyloric region.

(2) An ulcer in the pyloric (not the cardiac or fundus) region which produces stenosis is almost invariably accompanied by an abundant supersecretion—the result of acid catarrh.

(3) Statistics obtained by autopsy show that 86% of gastric ulcers present evidence of carcinomatous metaplasia; clinically, however, this can be demonstrated more frequently. The importance of early diagnosis is therefore self-evident.

(4) In pyloric obstruction the result of a simple ulcer, the removal (resection or gastro-enterostomy) does not relieve the supersecretion which may instead continue for quite a while (months, and at times, years).

(5) A pyloric ulcer which produces stenosis and is beginning to undergo cancerous change, may be recognized by a decrease in the aforesaid supersecretion, (harbinger of the future catarrhal state of the mucous membrane) as well as by a certain diminution in the secretion of gastric juice—secretory insufficiency.

(6) To demonstrate this decrease of superescretion (in cases of established stenosis), the gastric function is tested three times within the course of one day. Examination is made of the contents of the early morning stomach, that after a test breakfast and finally a test dinner.

"My observations have proved," says the author, "that, if in pyloric stenosis a supersecretion be found constantly after the three tests, we are perfectly justified in pronouncing the same a simple ulcer. Should there be found however a diminution, or total absence of free hydrochloric acid following any of these tests, we have cause to believe that in such a case, carcinoma has made its appearance. This rule holds good even if tests made at other isolated times, have shown a sufficient hydrochloric acid content. If necessary this examination should be repeated for a few days.—*Vermont Medical Monthly.*

